# Diversity and biological activity of freshwater copepodamides during a spring bloom in the Římov reservoir

**DOI:** 10.1093/ismeco/ycag166

**Published:** 2026-06-15

**Authors:** Gobardhan Sahoo, Petra Urajová, Jatishwor Singh Irungbam, Manickam Gayathri, Yaprak Şahin Karagöz, Divya Singh, Petr Znachor, Pavel Rychtecký, Jaromír Sed’a, Miloslav Devetter, Hannah C Knappe, Erik Selander, Rohit Ghai, Kumar Saurav

**Affiliations:** Laboratory of Algal Biotechnology, Centre Algatech, Institute of Microbiology of the Czech Academy of Sciences, Novohradská 237, Třeboň 37901, Czech Republic; Department of Ecology and Environmental Sciences, Pondicherry University, Puducherry 605014, India; Laboratory of Algal Biotechnology, Centre Algatech, Institute of Microbiology of the Czech Academy of Sciences, Novohradská 237, Třeboň 37901, Czech Republic; Laboratory of Algal Biotechnology, Centre Algatech, Institute of Microbiology of the Czech Academy of Sciences, Novohradská 237, Třeboň 37901, Czech Republic; Laboratory of Algal Biotechnology, Centre Algatech, Institute of Microbiology of the Czech Academy of Sciences, Novohradská 237, Třeboň 37901, Czech Republic; Laboratory of Algal Biotechnology, Centre Algatech, Institute of Microbiology of the Czech Academy of Sciences, Novohradská 237, Třeboň 37901, Czech Republic; Faculty of Science, University of South Bohemia, Branišovská 1760, České Budějovice 37005, Czech Republic; Laboratory of Algal Biotechnology, Centre Algatech, Institute of Microbiology of the Czech Academy of Sciences, Novohradská 237, Třeboň 37901, Czech Republic; Faculty of Science, University of South Bohemia, Branišovská 1760, České Budějovice 37005, Czech Republic; Faculty of Science, University of South Bohemia, Branišovská 1760, České Budějovice 37005, Czech Republic; Institute of Hydrobiology, Biology Centre of the Czech Academy of Sciences, Na Sádkách 7, České Budějovice 370 05, Czech Republic; Institute of Hydrobiology, Biology Centre of the Czech Academy of Sciences, Na Sádkách 7, České Budějovice 370 05, Czech Republic; Institute of Hydrobiology, Biology Centre of the Czech Academy of Sciences, Na Sádkách 7, České Budějovice 370 05, Czech Republic; Institute of Soil Biology and Biogeochemistry, Biology Centre of the Czech Academy of Sciences, Na Sádkách 7, České Budějovice 370 05, Czech Republic; Functional Ecology Section – Department of Biology, Lund University, Kontaktvägen 10, Lund 223 62, Sweden; Functional Ecology Section – Department of Biology, Lund University, Kontaktvägen 10, Lund 223 62, Sweden; Institute of Hydrobiology, Biology Centre of the Czech Academy of Sciences, Na Sádkách 7, České Budějovice 370 05, Czech Republic; Laboratory of Algal Biotechnology, Centre Algatech, Institute of Microbiology of the Czech Academy of Sciences, Novohradská 237, Třeboň 37901, Czech Republic

**Keywords:** copepods, copepodamides, freshwater plankton, Římov reservoir, molecular networking

## Abstract

Copepodamides are kairomones released by copepods, one of the most dominant zooplankton groups in aquatic ecosystems, which trigger anti-grazing defences in phytoplankton. Whilst widespread in marine ecosystems, their prevalence, diversity, and ecological roles in freshwater ecosystems remain largely unexplored. In this study, we conducted a comprehensive analysis of copepodamides from the eutrophic Římov Reservoir (Czech Republic) during the spring bloom to evaluate their diversity, temporal dynamics, and bioactivity. Using untargeted mass spectrometry, we identified 18 distinct dihydrocopepodamides (dhCAs), including five previously unreported variants. Interestingly, the congener 20:1 dhCA was detected in freshwater for the first time. Temporal analysis revealed that whilst 20:1 dhCA was initially restricted to the macroplankton fraction (>50 μm), its concentration in the microplankton fraction (<50 μm) gradually increased from 0% to 13% of total copepodamides during bloom progression, indicating active release or cellular turnover. Bioassays using the marine diatom *Skeletonema marinoi* demonstrated that the freshwater zooplankton extract containing dhCAs induced a concentration-dependent reduction in chain length, confirming the conservation of anti-grazing activity across aquatic habitats. However, it remains unclear whether freshwater microalgal species possess the molecular machinery to sense and respond to copepodamides. Overall, future studies on the effect of these kairomones on native phytoplankton species are essential to assess the ecological relevance of these compounds. This study represents one of the first systematic reports of freshwater copepodamides and highlights their potential role in shaping planktonic interactions and evolutionary trajectories in freshwater ecosystems.

Phytoplankton assemblages in freshwater ecosystems play an essential role in the food web dynamics and carbon cycle. However, their unchecked proliferation can lead to nuisance blooms, posing threats to human health and aquatic wildlife [1, 2]. Over the past few decades, the increasing frequency and severity of these blooms have become a growing concern [2–4]. Notably, bloom-forming phytoplankton, which are often responsible for the characteristic green colouration of eutrophic waters, can be influenced by zooplankton grazers and the kairomones they release [5, 6]. Amongst such interactions, copepods are known to secrete kairomones termed copepodamides, which imprint the water column with chemical cues (Fig. S1) [7]. These kairomones trigger a suite of defensive responses in marine phytoplankton, including phenotypic plasticity, toxin production, enhanced bioluminescence, and reinforced cell walls [8–10]. To date, such copepodamide-induced responses have been documented mainly in marine environments, except for six Swedish lakes [11], leaving a critical gap in our understanding of freshwater plankton ecology. In this context, the present study investigates temporal variation in copepodamide congeners in the eutrophic Římov Reservoir (Czech Republic) and provides evidence of their bioactivity using the marine model diatom *Skeletonema marinoi*.

Surface zooplankton and water samples from the Římov Reservoir were analyzed to relatively quantify copepodamide concentrations in two size fractions: the zooplankton fraction (>50 μm) and the microplankton fraction (<50 μm). Sampling was conducted three times per week over a 6-week period during March and April 2024, guided by chlorophyll a dynamics in the water column (Fig. 1a). The copepod community was predominantly composed of *Cyclops vicinus, Cyclops strenuus*, and *Megacyclops* species, followed by *Eudiaptomus gracilis* and *Mesocyclops leuckarti* (Fig. 1b). In total, 18 copepodamide variants were identified, all classified within the dihydrocopepodamides (dhCAs) subgroup and distinguished by diverse fatty acyl compositions (Fig. 1c and d). Amongst them, dhCA 17:2, dhCA 18:0, dhCA 19:5, dhCA 21:5, and dhCA 22:4 represent novel congeners documented for the first time in this study (Table S1). These findings align with previous observations indicating that dhCAs with even-numbered fatty acids tend to dominate freshwater ecosystems, even though odd-numbered variants are more prevalent in limnic systems compared to marine counterparts [11]. Although the biosynthetic mechanisms of copepodamides remain largely unknown, it is hypothesized that variability in their fatty acid composition is influenced by the dietary sources available to copepods in their environment [11, 12]. Notably, this study presents the first report of dhCA 20:1 in a freshwater setting, with its relative concentration increasing steadily from below detectable levels at the start of the sampling period to 13% by the end (Fig. 1d).

**Figure 1:**
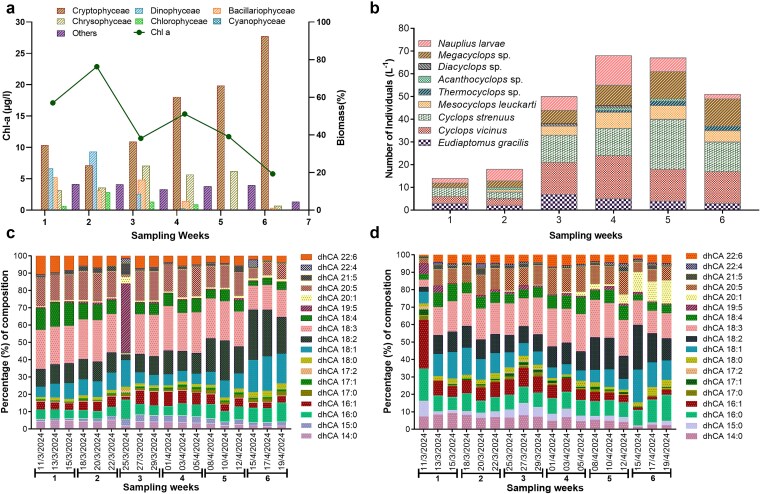
Temporal dynamics in the composition of (a) phytoplankton composition and chlorophyll-a, (b) copepods, (c) macroplankton fraction (>50 μm) dhCA, and (d) microplankton fraction (<50 μm) dhCA in Římov Reservoir, Czech Republic. The dhCA congeners were identified based on the diagnostic peak (m/z 432.278) and associated fragments (m/z 124.0; taurine) and (m/z 80; sulfonate) using Bruker Data Analysis software, ver. 6. Phytoplankton and copepod samples were collected weekly (Week 1: 11 March 2024, Week 2: 18 March 2024, Week 3: 25 March 2024, Week 4: 1 April 2024, Week 5: 8 April 2024, and Week 6: 15 April 2024). Copepodamide samples were collected three times weekly.

The ecological role, if any, of copepodamides in freshwater ecosystems remains unknown, but their consistent presence in freshwater copepods warrants experimental evaluation of their possible role as kairomones in these systems as well. Metabolomic profiling of phytoplankton biomass revealed temporal shifts, including the emergence of novel metabolite clusters during the final week of the sampling campaign (Fig. 2a; Fig. S4). Pearson correlation analysis between dhCA 20:1 and phytoplankton biomass showed a notably strong positive relationship (r = 0.86; *P* = .0268), specifically with the cryptophyte class Cryptophyceae (Table S2). Notably, *Cryptomonas reflexa* was abundant during the sampling period (Fig. 1a), a species well known for its high nutritional quality and commonly preferred by freshwater copepods [13]. These findings open intriguing avenues for future research, particularly on whether copepodamides modulate the defensive traits of freshwater copepods’ prey.

**Figure 2:**
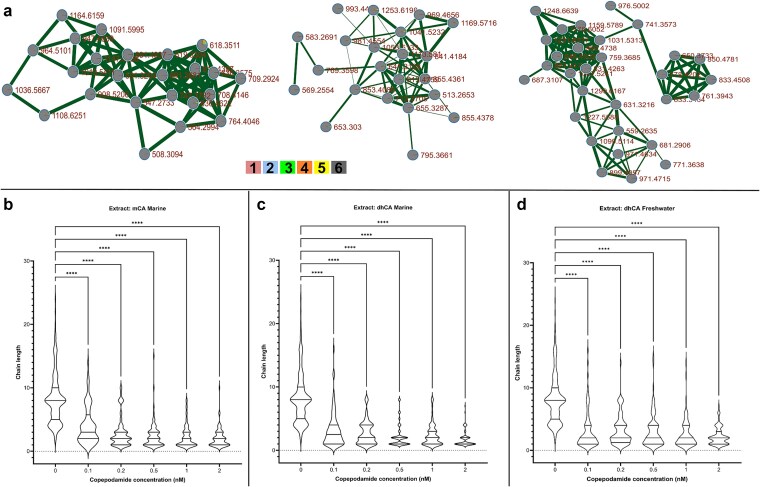
(a) Three clusters obtained from molecular network analysis at the sixth week of sampling represent distinct classes of metabolites, reflecting shifts in the phytoplankton community by the end of the spring sampling period in the Římov Reservoir (Czech Republic). Each node corresponds to an individual metabolite, labelled by its respective *m/z* value, and node colours indicate the different weeks of sampling. (b–d) Morphological response of *Skeletonema marinoi* to the (b) marine methylene-copepodamides (mCA), (c) marine dihydrocopepodamides (dhCA), and (d) freshwater dhCA extracts from the Římov Reservoir, Czech Republic. The marine mCA and dhCA extracts are the purified cocktails of different variants of copepodamides extracted from the marine copepod *Calanus finmarchicus*, whereas freshwater dhCA is from a crude methanol extract of zooplanktons containing all extractable metabolites. ^****^ indicates significant differences between the treatment group and control group (*P* < .0001).

Although freshwater copepodamides appear chemically identical to their marine analogues, their ecological function has yet to be evaluated. To investigate potential bioactivity, we exposed the marine diatom *Skeletonema marinoi*, a species known to respond to copepodamides by shortening its chain length [12], to zooplankton extract containing a mixture of dihydrocopepodamide (dhCA) congeners. Upon treatment, *S. marinoi* exhibited a dose-dependent morphological response, with chain length reduction becoming pronounced at a dhCA concentration of 2 nM, at which single cells dominated the population (Fig. 2b–d). This morphological shift is considered an adaptive trait to reduce susceptibility to predation [14–16]. Given that the freshwater extract used was a crude mixture of metabolites, additional parallel assays employing purified marine-derived copepodamides (mCAs and dhCAs) were performed. These confirmed comparable chain-shortening responses in *S. marinoi*, further supporting the hypothesis of functional conservation across marine and freshwater copepodamides (Fig. 2b and c).

These findings suggest that freshwater copepodamides are indeed bioactive and can exert effects on coexisting phytoplankton prey comparable to those of their marine counterparts. In freshwater systems, copepods have been shown to elicit colony formation in *Scenedesmus obliquus*, inhibit encystment in *Peridinium aciculiferum*, and stimulate microcystin production in *Microcystis aeruginosa* [6, 17, 18]. However, the specific chemical cues responsible for these responses remain unidentified. Toxic strains of *M. aeruginosa* and *Cylindrospermopsis raciborskii* producing microcystin and saxitoxin, respectively, exhibit reduced vulnerability to grazing by the calanoid copepod *E. gracilis* when compared to their non-toxic counterparts [19, 20]. This suggests a compelling link between copepod-mediated chemical signalling and the induction of toxin biosynthesis in phytoplankton prey. Elucidating the chemical basis of these interactions could provide deeper insights into the evolution of phytoplankton toxicity, an enduring question in aquatic ecology. Furthermore, the complex shifts observed within the phytoplankton community likely reflect multifactorial influences, including other grazers such as rotifers and cladocerans, as well as fluctuating environmental conditions (Fig. 1a, Figs S2 and S3) [21]. These dynamics reinforce the need for continued exploration into the ecological roles of copepodamides in limnic systems.

To our knowledge, this study represents the first experimental evaluation of dhCA bioactivity in a freshwater ecosystem. Continued research into these kairomones may provide critical insights into the nuanced mechanisms underlying prey–predator interactions and chemical communication amongst freshwater plankton. The emergence of a distinct set of new metabolites, alongside the sudden release of specific copepodamides, suggests a possible role for these compounds in driving metabolite production. Whilst various environmental and biological factors may contribute to this shift, our hypothesis is that copepodamides, acting as kairomones, may play a regulatory role in modulating phytoplankton metabolism.

## Supplementary Material

Sahoo_et_al_SI_June2026_ycag166

## Data deposition and job accessibility

The molecular networking job can be publicly accessed at https://gnps2.org/status?task=c7c073f99dec4b15aeea2b96e332dc2c#.
