# Supplementary material for: Diversity and biological activity of freshwater copepodamides during a spring bloom in the Římov reservoir
Source: ISME Commun. 2026 Jun 15;6(1):ycag166. doi: 10.1093/ismeco/ycag166 (PMC13440300; doi:10.1093/ismeco/ycag166)
Supplement: Supplementary file 1 [file sahoo_et_al_si_june2026_ycag166.docx]

**Diversity and Biological Activity of Freshwater Copepodamides During a Spring Bloom in the Římov Reservoir**

Gobardhan Sahoo^1, 2^, Petra Urajova^1^, Jatishwor Singh Irungbam^1^, Gayathri Manickam^1^, Yaprak Sahin^1,3^, Divya Singh^1,3^, Petr Znachor^3,4^, Pavel Rychtecký^4^, Jaromir Sed’a^4^, Miloslav Devetter^5^, Hannah C. Knappe^6^, Erik Selander^6^, Rohit Ghai^4^ and Kumar Saurav^1*^

**^1^**Laboratory of Algal Biotechnology, Centre Algatech, Institute of Microbiology of the Czech Academy of Sciences, Novohradská 237, Třeboň- 37901, Czech Republic

^2^Department of Ecology and Environmental Sciences, Pondicherry University, Puducherry-605014, India

**^3^**University of South Bohemia, Faculty of Science, Branišovská 1760, České Budějovice- 37005, Czech Republic

^4^Institute of Hydrobiology, Biology Centre Czech Academy of Sciences, Na Sádkách 7, České Budějovice- 370 05, Czech Republic

^5^Institute of Soil Biology and Biogeochemistry, Biology Centre Czech Academy of Sciences, Na Sádkách 7, České Budějovice- 370 05, Czech Republic

**^6^**Functional Ecology section - Department of Biology, Lund University, Kontaktvägen 10,

Lund- 223 62, Sweden

^*^Address correspondence to Kumar Saurav, sauravverma17@gmail.com.

Laboratory of Algal Biotechnology, Centre Algatech, Institute of Microbiology of the Czech Academy of Sciences, Novohradská 237, Třeboň-37901, Czech Republic

Running head: **Bioactive copepodamides from a freshwater reservoir**

**Contents:**

1. **Supplementary Methods**
2. **Supplementary Results**
3. **Supplementary Tables**
4. **Supplementary References**
1. **Supplementary Methods**
   1.1. ***Study site and sampling***

The Římov Reservoir, located in South Bohemia, Czech Republic, serves as a primary source of freshwater and was selected as the sampling site for this study. It is a canyon-shaped, dimictic, meso-eutrophic system with maximum and average depths of 43 m and 16 m, respectively [1]. Due to sustained phosphorus input, the reservoir exhibits strong longitudinal nutrient gradients, particularly during summer months when cyanobacterial blooms are frequently observed in its upstream regions [2-3]. This site has been subject to long-term ecological monitoring, with numerous studies documenting changes driven by both natural processes and anthropogenic pressures [1, 4]. For the present investigation on copepodamides, zooplankton and surface water samples were collected thrice weekly, on Mondays, Wednesdays, and Fridays, over a six-week period during March and April 2024. The sampling schedule was guided by fluctuations in surface chlorophyll a concentration, which served as a proxy for phytoplankton biomass and bloom dynamics.

1.2. ***Analysis of environmental variables***

Surface water temperature, pH, and dissolved oxygen concentrations were measured in situ using a portable multiparametric probe (YSI EXO 2, Yellow Springs Instruments, USA). Nutrient samples, including silicate, reactive phosphorus, nitrate, nitrite, and ammonia, were collected from surface water and quantified spectrophotometrically following established protocols [5-6]. Water transparency was determined using a 30 cm diameter Secchi disk. To estimate chlorophyll a concentration, water samples were filtered through Whatman GF/C filters (pore size 1 µm), then extracted with ethanol and analysed spectrophotometrically according to ISO 10260 (1992).

1.3. ***Zooplankton sampling***

Zooplanktons were sampled by vertical hauls using an Apstein plankton net (200 μm mesh size). Two vertical hauls were taken from 5 m to the surface (755 L in total), representing the depth of the epilimnion. For taxonomic analysis, 40 L of sampled water were collected once weekly and were subsequently concentrated using a 30 µm net. The samples were preserved in 4% formalin, and the abundance of the zooplankton taxa was microscopically identified following a previously described method [7] with copepods identified to the species level wherever possible.

For copepodamide analysis, 40 L of sampled water was concentrated to 1 L using a 30 µm net and immediately transported to the laboratory. Upon arrival, it was passed through a 50 µm sieve to separate two size fractions: the zooplankton fraction (>50 µm) and the microplankton fraction (<50 µm). The retained material (residue) and exactly 40 mL of the filtrate were collected separately for copepodamide (CA) extraction to enable relative quantification. All samples were stored at −20 °C until further processing. Samples were later lyophilised using a ScanVac CoolSafe Touch system (LaboGene A/S, Allerød, Denmark) and soaked in 100% methanol (MeOH). Zooplankton samples were incubated overnight at 4°C for efficient CA extraction from the biomass, while water samples were processed immediately. Both sample types were sonicated and centrifuged (4°C, 5000 rpm, 10 min). The supernatant was collected and concentrated using a stream of N₂ vapor at 40°C, then redissolved in 1 mL MeOH. An aliquot was analysed on High-Pressure Liquid Chromatography connected to a High-Resolution Mass Spectrometer (HPLC-HRMS) for the detection of CAs. The extract derived from zooplankton biomass—hereafter referred to as freshwater dhCA extract was stored at −20°C, and its bioactivity was subsequently tested using the marine diatom *Skeletonema marinoi* as a positive control.

1.4. ***Phytoplankton sampling for taxonomic composition and metabolites***

Surface water for phytoplankton analysis was collected weekly and immediately fixed with Lugol’s solution at a final concentration of 1%. Phytoplankton were microscopically identified to the lowest possible taxonomic level following the previously described method [8]. Cell dimensions were measured by comparing their morphology to standard geometrical shapes to calculate biovolume, as outlined before [9]. For metabolite composition analysis, water samples collected after zooplankton removal were filtered through a 20 µm phytoplankton net and transported to the laboratory. Sampling was conducted three times per week. Upon arrival, samples were immediately frozen and stored until further processing.

1.5. ***Detection of Copepodamides by High-Resolution Mass Spectrometry (HRMS)***

An aliquot (20 µl) of the sample was injected into HPLC (Thermo Scientific Dionex UltiMate 3000) equipped with a diode-array detector (DAD) and coupled with high-resolution mass spectrometry with electrospray ionisation source (ESI-HRMS; Impact HD Mass Spectrometer, Bruker) for the detection of CAs. The elution of CAs was carried out by an Amide column (TSK gel Amide-80, 5 µm, 4.6 mm x 25 cm) in the UHPLC with MeOH (eluent A) and H_2_O (eluent B) as mobile phase at a constant flow of 0.6 mL min^-1^. Both the eluents were acidified (0.1%) before use. Initially, the solvent gradient was maintained with 98% eluent A for 3 min. Then, a linear increase in eluent B to 60% over 22 min was performed, followed by a sharp increase to 95% for 1 min, then held for 4 min. Subsequently, the concentration of eluent A was increased to 98% over 1 min, then held at that level for 2 min before re-equilibrating the column with 2% eluent B. After the elution, CAs were detected in HRMS operated in negative ionisation with the following settings: dry temperature 250°C, drying gas flow 8 L/min, nebuliser pressure 1 bar, capillary voltage 2500 V, and endplate offset 500 V. The collision-induced dissociation (CID) ramp was altered from 40 to 60 eV for masses 500 -1000 *m/z*, respectively. Calibration was performed using CH_3_COONa clusters at the beginning of each analysis.

The spectra were collected in the range of 20-1000 *m/z* at a rate of 3 Hz, and the presence of CAs was checked in the extraction ion chromatogram in all MSn spectra (EIC) using Bruker Data Analysis software, ver. 6.1. The CAs have primary fragments with m/z 430.263 and 432.278, which are fingerprints of subgroups: methylene-copepodamides (mCAs; C_22_H_40_NO_5_S) and dihydrocopepodamides (dhCAs; C_22_H_42_NO_5_S), respectively [10]. After the detection of these fragment ions in MSMS spectra, the presence of additional fragments characteristic of copepodamides with m/z 124.0 (taurine; C_2_H_6_NO_3_S) and m/z 80 (sulfonate; SO_3_) was checked. Then, the peak areas of molecular ions associated with CAs were calculated for each sample using Bruker TASQ software 2024b.

**Fig. S1**. General structure of a copepodamide (R^1^ = -methyl (dhCA) or -methylene group (mCA)) (Adapted from Arnoldt et al., 2024) [11]. R^2^ = -variable acyl chain and are quite variable with diet.

1.6. ***Metabolomic analysis of phytoplankton***

The phytoplankton samples were lyophilized, and their dry weight was noted down. The metabolites from these samples were extracted in 70% MeOH in water, followed by bath sonication. A Thermo Scientific Dionex UltiMate 3000 UHPLC (Thermo Fisher Scientific, Waltham, MA, USA) equipped with a diode-array detector (DAD) and high-resolution mass spectrometry with an electrospray ionization source (ESI-HRMS; Impact HD Mass Spectrometer, Bruker Billerica, MA, USA) was used for the analysis of the crude extracts. HPLC separation was performed on a reversed-phase Kinetex Phenomenex C_18_ column (150 × 4.6 mm, 2.6 µm; Phenomenex, Aschaffenburg, Germany) with H_2_O/acetonitrile containing 0.1% HCOOH as a mobile phase. The flow rate during the analysis was 0.6 mL/min. The gradient was as follows: H_2_O/ACN 85/15 (0 min), 85/15 (1 min), 0/100 (20 min), 0/100 (25 min), and 85/15 (30 min). The mass spectrometer operated in positive ionisation and the settings were as follows: dry temperature 200 °C; drying gas flow 10 L/min; nebulizer 3 bar; capillary voltage 4500 V; endplate offset 500 V. The spectra were collected over the range 20–2000 *m/z,* with a scan rate of 4 Hz. A ramp was set with collision-induced dissociation from 20 to 60 eV on successive *m/z* 200–1200. Calibration was performed using CH_3_COONa clusters at the beginning of each analysis. The raw data files obtained from HPLC-HRMS/MS analysis were converted to mzXML format using MSConvert from the ProteoWizard suite [12]. A molecular network was created with the Feature-Based Molecular Networking (FBMN) workflow on GNPS2 ([GNPS2 - Analysis Hub](https://gnps2.org/homepage)) [13-14]. The mass spectrometry data were first processed with MZMINE3, and the results were exported to GNPS for FBMN analysis [15]. The data were filtered by removing all MS/MS fragment ions within +/- 17 Da of the precursor m/z. MS/MS spectra were window filtered to retain only the top 6 fragment ions in the +/- 50 Da window throughout the spectrum. The precursor ion mass tolerance was set to 0.02 Da, and the MS/MS fragment ion tolerance to 0.02 Da. A molecular network was then created, with edges filtered to have a cosine score above 0.7 and more than 5 matched peaks. Further, edges between two nodes were kept in the network if and only if each node appeared in the other's top 10 most similar nodes. Finally, the maximum molecular family size was set to 100, and the lowest-scoring edges were removed from molecular families until the molecular family size fell below this threshold. The analogue search mode was used by searching against MS/MS spectra with a maximum difference of 100.0 in the precursor ion value. The library spectra were filtered in the same manner as the input data. All matches kept between network spectra and library spectra were required to have a score above 0.7 and at least 6 matched peaks. The DEREPLICATOR was used to annotate MS/MS spectra [16]. Additional edges were provided by the user. The molecular networks were visualized using Cytoscape software [17].

1.7. ***Procurement of Copepodamides (CAs) extracted from the marine copepod Calanus finmarchicus***

Marine methylene-copepodamides (mCAs) and dihydrocopepodamides (dhCAs) used in this investigation were extracted from the marine copepod *Calanus finmarchicus* as described in Selander et al 2015.

1.8. ***Bioactivity assay for freshwater zooplankton extract containing dhCAs***

Due to limited knowledge of the functional role of freshwater dhCAs, a bioassay was conducted to confirm their activity on the marine diatom *Skeletonema marinoi*, which served as a positive control. Exponential cultures of *S. marinoi* growing in f/2 medium (+silicate) were exposed to five nominal concentrations of zooplankton extract containing dhCAs: 0.1nM, 0.2nM, 0.5nM, 1nM, and 2nM. This zooplankton extract was prepared from crude zooplankton and contained all the metabolites, including dhCA. Hence, parallel assays using purified marine mCA and dhCA extracts were also performed to distinguish the effects of freshwater dhCAs from those of other metabolites. These assays were conducted in glass vials (volume: 8.7ml) with replicates (N=4). Prior to transferring cultures, the vials were coated with an extract containing mCAs or dhCAs at nominal concentrations, then left to evaporate the MeOH for approximately 30 minutes. Separately, a set of controls was also used in which only MeOH was added. This method was previously used and have shown that the average effective concentration of CAs in solution is around 0.1-1.5% of the nominal concentrations due to dissolution and degradation processes [18]. Thus, the final concentrations of CAs to which the diatom was exposed were in picomolar ranges, making the bioassay ecologically relevant. All the vials were loaded onto a plankton wheel (1-2 rotations min^-1^), and the bioassay was continued for 72h under optimum conditions (16˚C temperature, 100 µmol m-2 s-1 white light intensity, and 16:8 light:dark photoperiod). After the end of the experiment, the diatoms were observed under the microscope, and 30 random chains per condition were counted. Since the data were not normally distributed (Shapiro-Wilk test), a nonparametric Kruskal-Wallis test followed by a post hoc test was performed using GraphPad Prism version 10.0.0. The variability between the groups was considered significant when p<0.05.

2. **Supplementary Results**
   2.1. ***Environmental factors***

There was little fluctuation in environmental factors, including Secchi depth (m), pH, and dissolved oxygen (mg/L) in the surface water of the Římov reservoir during the sampling period (Figure S2). Among the nutrients, dissolved silicate (mg/L) concentration also remained almost stable, whereas soluble reactive phosphorus and total nitrogen exhibited minor variations ranging from 8.8 to 17.6 µg/L and 1.20 to 1.66 mg/L, respectively (Figure S2).

**Figure S2**. Temporal dynamics of environmental factors over a period of 6 weeks at Římov Reservoir, Czech Republic

2.2. ***Zooplankton composition***

The zooplankton abundance increased gradually from 28 ind. L^-1^ in the first week of sampling to 335 ind. L^-1^ in the fifth week and decreased to 219 ind. L^-1^ (Figure S3). Among the groups, copepods dominated the community in the first week and showed a gradual increase in abundance until the fifth week, after which they slightly decreased in the sixth week. In contrast, rotifers' abundance was low in the first week, but they outnumbered other groups thereafter. The cladoceran number was low initially, with intermittent high abundance (fifth week). On the other hand, ostracods were occasionally found, but only at very low abundance (Figure S3).

**Fig. S3**. Variability in the zooplankton composition over a period of 6 weeks at Římov Reservoir, Czech Republic

2.3. ***Phytoplankton metabolites analysis***

The LC-MS/MS data from phytoplankton extracts were processed using the GNPS platform, yielding a feature-based molecular network that revealed distinct clusters of structurally related metabolites. The feature-based molecular networking (FBMN) analysis revealed a dynamic and temporally structured metabolomic profile across the phytoplankton sampling period. The network comprised 1553 Nodes and 2509 Edges, indicating a diverse array of compounds with shared fragmentation patterns. Several major clusters were observed, corresponding to known metabolite families such as Mycosporine-like amino acids (MAAs), detected in clusters with high spectral similarity and matched to GNPS library entries, and Polyunsaturated fatty acid derivatives forming two dense subnetworks, suggesting biosynthetic relatedness (Figure S4). A distinct onset of new molecular clusters was observed toward the end of the sampling timeline, indicating the biosynthesis of an entirely new class of compounds (Figure 2A). This emergence strongly suggests the influence of external environmental factors, particularly changes in the composition or concentration of copepodamides, which are known to modulate phytoplankton metabolism. Such findings highlight the ecological sensitivity of phytoplankton metabolite production and underscore the utility of FBMN in capturing chemically responsive shifts driven by biotic interactions in aquatic ecosystems.

**Figure S4**. A feature-based molecular network (1,553 nodes; 2,509 edges) was generated from HRMS/MS data of phytoplankton extracts using GNPS2. Each node represents a metabolite characterized by its m/z value and fragmentation pattern, while edges indicate spectral similarity and group related compounds into clusters. Metabolites were annotated using GNPS spectral libraries. Node colour represents the sampling week, enabling visualization of temporal variation in metabolite occurrence. Clusters correspond to chemically related metabolite families, and changes in node distribution and colour composition across clusters highlight shifts in metabolite profiles over time. These patterns reflect the dynamic succession of the phytoplankton community during the sampling period.

3. **Supplementary tables**

**Table S1.** List of copepodamides detected in Římov reservoir, Czech Republic. The fatty acid chain lengths ranged from C_14_ to C_22_ with degrees of unsaturation from 0 to 5.

| **Copepodamide** | **Mass (Da)** | **formula** | **Reported occurrence in** |
| --- | --- | --- | --- |
| dhCA 14:0 | 661.4951 | C_36_H_71_NO_7_S | Marine, Freshwater |
| dhCA 15:0 | 675.5108 | C_37_H_73_NO_7_S | Marine, Freshwater |
| dhCA 16:0 | 689.5264 | C_38_H_75_NO_7_S | Marine, Freshwater |
| dhCA 16:1 | 687.5108 | C_38_H_73_NO_7_S | Marine, Freshwater |
| dhCA 17:0 | 703.5421 | C_39_H_77_NO_7_S | Marine, Freshwater |
| dhCA 17:1 | 701.5264 | C_39_H_75_NO_7_S | Marine, Freshwater |
| dhCA 17:2 | 699.5108 | C_39_H_73_NO_7_S | Freshwater, this study |
| dhCA 18:0 | 717.5577 | C_40_H_79_NO_7_S | Freshwater, this study |
| dhCA 18:1 | 715.5421 | C_40_H_77_NO_7_S | Marine, Freshwater |
| dhCA 18:2 | 713.5264 | C_40_H_75_NO_7_S | Marine, Freshwater |
| dhCA 18:3 | 711.5108 | C_40_H_73_NO_7_S | Marine, Freshwater |
| dhCA 18:4 | 709.4951 | C_40_H_71_NO_7_S | Marine, Freshwater |
| dhCA 19:5 | 721.4951 | C_41_H_71_NO_7_S | Freshwater, this study |
| dhCA 20:1 | 743.5734 | C_42_H_81_NO_7_S | Marine, Freshwater, this study |
| dhCA 20:5 | 735.5108 | C_42_H_73_NO_7_S | Marine, Freshwater |
| dhCA 21:5 | 749.5264 | C_43_H_75_NO_7_S | Freshwater, this study |
| dhCA 22:4 | 765.5577 | C_44_H_79_NO_7_S | Freshwater, this study |
| dhCA 22:6 | 761.5264 | C_44_H_75_NO_7_S | Marine, Freshwater |

**Table S2**. Statistical results of Pearson correlation between the relative abundance of dhCA 20:1 and the relative biomass of phytoplankton taxa.

| Correlation between variables | Correlation coefficient (r) | p value |
| --- | --- | --- |
| dhCA 20:1 and Cryptophyceae | 0.86 | 0.03 |
| dhCA 20:1 and Dinophyceae | -0.52 | 0.29 |
| dhCA 20:1 and Bacillariophyceae | -0.62 | 0.19 |
| dhCA 20:1 and Chrysophyceae | -0.65 | 0.16 |
| dhCA 20:1 and Chlorophyceae | -0.56 | 0.24 |
| dhCA 20:1 and Cyanophyceae | 0.72 | 0.11 |
| dhCA 20:1 and Chlorophyll-a | -0.79 | 0.06 |

4. **References**

1.	Znachor, P.; Nedoma, J.; Hejzlar, J.; Seďa, J.; Kopáček, J.; Boukal, D., et al., Multiple long-term trends and trend reversals dominate environmental conditions in a man-made freshwater reservoir. *The Science of the total environment* 2018**,** **624**, 24-33. <https://doi.org/10.1016/j.scitotenv.2017.12.061>

2.	Rychtecký, P.; Znachor, P., Spatial heterogeneity and seasonal succession of phytoplankton along the longitudinal gradient in a eutrophic reservoir. *Hydrobiologia* 2011**,** **663** (1), 175-186. <https://doi.org/10.1007/s10750-010-0571-6>

3.	Znachor, P.; Visocká, V.; Nedoma, J.; Rychtecký, P., Spatial heterogeneity of diatom silicification and growth in a eutrophic reservoir. *Freshwater Biology* 2013**,** **58** (9), 1889-1902. <https://doi.org/10.1111/fwb.12178>

4.	Znachor, P.; Nedoma, J.; Hejzlar, J.; Seďa, J.; Komárková, J.; Kolář, V., et al., Changing environmental conditions underpin long-term patterns of phytoplankton in a freshwater reservoir. *The Science of the total environment* 2020**,** **710**, 135626. <https://doi.org/10.1016/j.scitotenv.2019.135626>

5.	Murphy, J.; Riley, J. P., A modified single solution method for the determination of phosphate in natural waters. *Analytica Chimica Acta* 1962**,** **27**, 31-36. <https://doi.org/10.1016/S0003-2670(00)88444-5>

6.	Procházková, L., Bestimmung der Nitrate im Wasser. *Fresenius' Zeitschrift für analytische Chemie* 1959**,** **167** (4), 254-260. <https://doi.org/10.1007/BF00458786>

7.	McCauley E, D. J., Rigler FH, *A manual on methods for the assessment of secondary productivity in fresh waters*. 1984.

8.	Lund, J. W. G.; Kipling, C.; Le Cren, E. D., The inverted microscope method of estimating algal numbers and the statistical basis of estimations by counting. *Hydrobiologia* 1958**,** **11** (2), 143-170. <https://doi.org/10.1007/BF00007865>

9.	Hillebrand, H.; Dürselen, C.-D.; Kirschtel, D.; Pollingher, U.; Zohary, T., Biovolume calculation for pelagic and benthic microalgae. *Journal of Phycology* 1999**,** **35** (2), 403-424. <https://doi.org/10.1046/j.1529-8817.1999.3520403.x>

10.	Grebner, W.; Berglund, E. C.; Berggren, F.; Eklund, J.; Harðadóttir, S.; Andersson, M. X., et al., Induction of defensive traits in marine plankton—new copepodamide structures. *Limnology and Oceanography* 2019**,** **64** (2), 820-831. <https://doi.org/10.1002/lno.11077>

11.	Arnoldt, S.; Pourdanandeh, M.; Spikkeland, I.; Andersson, M. X.; Selander, E., Mass spectroscopy reveals compositional differences in copepodamides from limnic and marine copepods. *Scientific Reports* 2024**,** **14** (1), 3147. <https://doi.org/10.1038/s41598-024-53247-1>

12.	Chambers, M. C.; Maclean, B.; Burke, R.; Amodei, D.; Ruderman, D. L.; Neumann, S., et al., A cross-platform toolkit for mass spectrometry and proteomics. *Nature Biotechnology* 2012**,** **30** (10), 918-920. <https://doi.org/10.1038/nbt.2377>

13.	Nothias, L.-F.; Petras, D.; Schmid, R.; Dührkop, K.; Rainer, J.; Sarvepalli, A., et al., Feature-based molecular networking in the GNPS analysis environment. *Nature Methods* 2020**,** **17** (9), 905-908. <https://doi.org/10.1038/s41592-020-0933-6>

14.	Wang, M.; Carver, J. J.; Phelan, V. V.; Sanchez, L. M.; Garg, N.; Peng, Y., et al., Sharing and community curation of mass spectrometry data with Global Natural Products Social Molecular Networking. *Nature Biotechnology* 2016**,** **34** (8), 828-837. <https://doi.org/10.1038/nbt.3597>

15.	Schmid, R.; Heuckeroth, S.; Korf, A.; Smirnov, A.; Myers, O.; Dyrlund, T. S., et al., Integrative analysis of multimodal mass spectrometry data in MZmine 3. *Nature Biotechnology* 2023**,** **41** (4), 447-449. <https://doi.org/10.1038/s41587-023-01690-2>

16.	Mohimani, H.; Gurevich, A.; Shlemov, A.; Mikheenko, A.; Korobeynikov, A.; Cao, L., et al., Dereplication of microbial metabolites through database search of mass spectra. *Nature Communications* 2018**,** **9** (1), 4035. <https://doi.org/10.1038/s41467-018-06082-8>

17.	Shannon, P.; Markiel, A.; Ozier, O.; Baliga, N. S.; Wang, J. T.; Ramage, D., et al., Cytoscape: A Software Environment for Integrated Models of Biomolecular Interaction Networks. *Genome Research* **13** (11), 2498-2504. <https://doi.org/10.1101/gr.1239303>

18.	Selander, E.; Berglund, E. C.; Engström, P.; Berggren, F.; Eklund, J.; Harðardóttir, S., et al., Copepods drive large-scale trait-mediated effects in marine plankton. *Science Advances* 2019**,** **5** (2), eaat5096. <https://doi.org/10.1126/sciadv.aat5096>
